# Prevalence and predictors of prolonged length of stay among patients admitted under general internal medicine in a tertiary government hospital in Manila, Philippines: a retrospective cross-sectional study

**DOI:** 10.1186/s12913-022-08885-4

**Published:** 2023-01-18

**Authors:** John Jefferson V. Besa, Ella Mae I. Masamayor, Diana R. Tamondong-Lachica, Lia M. Palileo-Villanueva

**Affiliations:** 1 Department of Medicine, College of Medicine, University of the Philippines Manila, Manila, Philippines; 2 Department of Medicine, University of the Philippines Manila - Philippine General Hospital, Manila, Philippines

**Keywords:** Health system quality, Healthcare delivery, Healthcare financing, Health policy, Patient care, Prolonged hospitalization, Internal medicine, Hospital medicine

## Abstract

**Background:**

Prolonged hospitalization leads to poorer health outcomes and consumes limited hospital resources. This study identified factors associated with prolonged length of stay (PLOS) among internal medicine patients admitted in a tertiary government hospital.

**Methods:**

We reviewed the medical records of 386 adult patients admitted under the primary service of General Internal Medicine at the Philippine General Hospital from January 1 to December 31, 2019. PLOS was defined as at least 14 days for emergency admissions or 3 days for elective admissions. Sociodemographics, clinical characteristics, admission- and hospital system-related factors, disease-specific factors, outcome on the last day of hospitalization, and hospitalization costs were obtained. We determined the proportion with PLOS and reviewed reasons for discharge delays. We conducted multiple logistic regression analyses to assess associations between various factors and PLOS.

**Results:**

The prevalence of PLOS is 19.17% (95% CI 15.54, 23.42). Positive predictors include being partially dependent on admission (aOR 2.61, 95% CI 0.99, 6.86), more co-managing services (aOR 1.26, 95% CI 1.06, 1.50), and longer duration of intravenous antibiotics (aOR 1.36, 95% CI 1.22, 1.51). The only negative predictor is the need for intravenous antibiotics (aOR 0.14, 95% CI 0.04, 0.54). The most common reason for discharge delays was prolonged treatment. The median hospitalization cost of patients with PLOS was PHP 77,427.20 (IQR 102,596).

**Conclusions:**

Almost a fifth of emergency admissions and a quarter of elective admissions had PLOS. Addressing factors related to predictors such as functional status on admission, number of co-managing services, and use of intravenous antibiotics can guide clinical and administrative decisions, including careful attention to vulnerable patients and judicious use of resources.

**Supplementary Information:**

The online version contains supplementary material available at 10.1186/s12913-022-08885-4.

## Background

Hospital length of stay is a key metric of hospital efficiency and quality of patient care [1, 2]. Prolonged hospitalization is associated with increased inpatient complications, poor patient outcomes, and high hospital expenditure [3–6]. Although less than a fifth of admitted patients were observed to have prolonged length of stay (PLOS), they utilized almost half of all hospital bed-days [7]. Associated factors vary across studies and include younger age, male sex, functional status, number of comorbidities, emergency hospitalizations, admission past 5 PM, weekend admissions, hospital-acquired infections, palliative care consults, need for a post-acute care facility, intensive care unit admissions, surgery, lower physician-to-patient ratio, and lower socioeconomic status [1, 3, 4]. Currently, there is no consensus definition for PLOS.

Identifying patients at risk for PLOS and other associated factors is a rational strategy to reduce hospital length of stay. Clinicians will be aware of patients with higher risk for complications and poorer outcomes while administrators will be able to create well-informed decisions in hospital management. This has not been investigated in lower-middle income countries which are resource-limited settings. This study determined the prevalence of PLOS, compared the sociodemographic and clinical characteristics of patients with normal length of stay (NLOS) and PLOS, identified factors associated with PLOS, described reasons for discharge delays, and estimated hospitalization costs of PLOS among general internal medicine patients admitted in a tertiary government hospital.

## Methods

### Study design

We performed a cross-sectional study through retrospective review of medical records of patients admitted at the emergency department, general medicine wards, and the medical intensive care unit of the Philippine General Hospital. The study protocol was approved by the University of the Philippines Manila Review Ethics Board (UPMREB 2020–506-01).

### Study setting

We conducted the study in the University of the Philippines—Philippine General Hospital (UP-PGH), a public teaching hospital and a national tertiary referral center in Manila, Philippines with a 1500-bed capacity [8].

The Department of Medicine is the largest clinical department in UP-PGH, with 13 subspecialty divisions. It includes both outpatient and inpatient services, either as service (i.e. fully subsidized) patients or private (Pay) patients. Service patients are admitted either directly to the wards or Medical Intensive Care Unit (MICU) or at the emergency department then transferred to the wards or the MICU. All admitted service patients are managed by the primary attending service, composed of the service consultant, senior resident-in-charge, junior resident-in-charge, and co-managing fellows-in-training. The department also receives referrals from other departments for co-management and preoperative evaluation which are assigned to a general internal medicine service.

### Study population

We included all hospital admissions to the General Internal Medicine service between January 1, 2019 to December 31, 2019 that fulfill the following inclusion criteria: age 19 years and older, admitted at least 24 h under the General Internal Medicine as a service patient regardless of area, and General Internal Medicine as the primary attending service on admission. The following patients were excluded: those under the primary service of other departments, those transferred to or from another service or department, or those transferred to or from the Pay services. For patients with multiple admissions, we considered each admission separately.

We classified eligible hospital admissions as either elective or emergency, according to the acuity of the reason for admission. Elective admissions were those directly admitted to the general medicine wards or MICU for non-urgent, elective procedures such as percutaneous coronary intervention, imaging-guided biopsy, and blood transfusions. Admissions through the emergency department for acute urgent or emergent problems that were eventually discharged directly from the ER or transferred to the wards or the MICU were considered emergency admissions.

### Study variables

Length of stay was defined as the time from the day of admission at the emergency room or wards to the last day of hospitalization. As per hospital policy, prolonged length of stay (PLOS) was defined as 14 days or longer for emergency admissions and 3 days or longer for elective admissions; otherwise, it was considered as normal length of stay (NLOS). We obtained the following variables on admission: age, sex, distance of place of residence from the hospital, highest educational attainment, employment status, Medical Social Service classification, smoking status, level of alcohol consumption, functional status, comorbidities, Charlson Comorbidity Index score, and history of prior hospitalization in the past 30 days. We also reviewed the records for the following variables: type of admission (emergency or elective), day of admission, time of admission, number of medications on admission, need for intravenous antibiotics and duration, duration of emergency room and intensive care unit stay, need for invasive and non-invasive ventilation and duration, performance of procedure and surgery, type of surgery and surgical risk of non-cardiac surgeries, need for blood transfusion, need for dialysis, development of shock, type, and duration, development of in-hospital complications and healthcare-associated infections, number of co-managing services, presence of signed advance directive, outcome of hospitalization, and cause of death, if applicable. Their corresponding operational definitions are detailed in Supplementary Table 1. Direct medical costs based on hospital bills, which excluded professional fees of health personnel, were also determined.

Reasons for delay in discharge were reviewed in the weekly census of overstaying patients. Two independent adjudicators classified them as administrative (e.g. delay in procedure schedules, lack of blood products), disease-related (e.g. completion of intravenous antibiotics, difficulty in weaning, need for workup, development of new medical problems, need for palliative care), or patient-related (e.g. home care issues, caregiver issues, financial issues). A third adjudicator was called in cases where the two independent reviewers had conflicting classifications.

### Sample size

Sample size was computed to be 344 using G*Power 3.1 with a 95% confidence level and a power of 0.8 using the odds ratio on risk factors for prolonged hospital stay [4, 9]. We used a simple random sampling method. All admissions that fulfilled the inclusion criteria were encoded in Microsoft Excel and assigned a random number through its random number generator function. The list was sorted in ascending order according to the random numbers generated and served as the study’s sampling frame. Eligible admissions were enrolled consecutively until the desired sample size was reached.

### Statistical analysis

Categorical data were expressed as frequencies and percentages while continuous variables were summarized using median and interquartile range. The median, interquartile range, minimum, and maximum of length of stay and direct medical costs and prevalence of PLOS were calculated. Characteristics of admissions of PLOS and NLOS were compared using t-test, chi-square test, Fisher’s exact test, and Mann–Whitney U test.

Multiple logistic regression analysis was used to evaluate the association of marginally associated variables on crude logistic regression analysis with p-values of at least < 0.25 and having frequencies of at least 5 on all cells. No imputation was done for missing data. The effect sizes from the multivariate analyses are reported as odds ratio. Confidence interval was set at 95%, and a p-value less than 0.05 was considered significant. STATA 16 was used for the analyses [10].

## Results

We randomly selected and reviewed the medical records of 386 out of 6,522 admissions under General Internal Medicine between January 1, 2019 to December 31, 2019. The prevalence of PLOS is 19.17% (95% CI 15.54, 23.42). Of all the admissions, 64 out of 347 (18%) emergency admissions and 10 out of 39 (26%) elective admissions had PLOS. The median length of stay among emergency and elective admissions with PLOS is 20.5 (IQR 8) days and 3.5 (IQR 3) days, respectively. The mean differences in length of stay between PLOS and normal length of stay (NLOS) among emergency and elective admissions is 18 days (95% CI 16.19, 19.85) for emergency and 8 days (95% CI 1.67, 14.42) for elective admissions.

Table 1 compares the characteristics of admissions with PLOS and NLOS. Those with PLOS had worse functional status, were more likely to have thyroid disease on admission, and needed more intravenous antibiotics, invasive and non-invasive ventilation, thoracentesis, central venous catheter insertion, surgeries, and blood transfusion than those with NLOS. They also had longer intensive care unit stays, more days on intravenous antibiotics, invasive ventilation, and non-invasive ventilation, and more co-managing specialty and subspecialty services. More in-hospital complications, specifically healthcare-associated infections (hospital-acquired pneumonia and ventilator-associated pneumonia), respiratory complications, and adverse drug events/adverse drug events occurred in admissions with PLOS. They also incurred higher hospitalization costs. The results of the rest of the variables are listed in Supplementary Table 2.

**Table 1:** Characteristics of admissions with normal and prolonged length of stay

Variable	PLOS (*n* = 74)	NLOS (*n* = 312)	*P*-value
Age, in years, mean (SD)	52.74 (14.14)	51.69 (14.85)	0.58^a^
Female, n (%)	27 (57.55%)	130 (41.67%)	0.42^b^
Employment status (*N* = 362)^c^			0.43^b^
Unemployed, n (%)	51 (71.83%)	195 (67.01%)	
Smoking status (*N* = 382)^c^			0.87^d^
Never, n (%)	39 (52.70%)	153 (52.70%)	
Previous, n (%)	16 (21.62%)	76 (24.68%)	
Current, n (%)	19 (25.68%)	79 (25.65%)	
Level of alcohol consumption (*N* = 388)^c^			0.09^d^
Never, n (%)	48 (71.64%)	246 (86.01%)	
Occasional, n (%)	13 (19.40%)	26 (9.09%)	
Heavy, n (%)	6 (8.96%)	14 (4.90%)	
Functional status (*N* = 359)^c^			0.02^d^
Independent, n (%)	48 (71.64%)	252 (86.30%)	
Partially Dependent, n (%)	13 (19.40%)	26 (8.90%)	
Totally Dependent, n (%)	6 (8.96%)	14 (4.79%)	
Number of comorbidities, median (IQR)	1 (2)	1 (2)	0.66^e^
Comorbidities			
Hypertension, n (%)	27 (36.49%)	122 (39.10%)	0.68^b^
Diabetes, n (%)	4 (5.41%)	15 (4.72%)	0.76^d^
Ischemic heart disease, n (%)	3 (4.05%)	10 (3.21%)	0.72^d^
Heart failure, n (%)	4 (5.41%)	23 (7.37%)	0.55^b^
Bronchial asthma, n (%)	4 (5.41%)	14 (4.49%)	0.75^d^
Thyroid disease, n (%)	4 (5.41%)	3 (0.96%)	0.03^d^
Chronic kidney disease, n (%)	12 (16.22%)	39 (12.50%)	0.40^b^
Malignancy/cancer, n (%)	11 (14.86%)	39 (12.50%)	0.59^b^
Charlson Comorbidity Index, median (IQR)	3 (3)	3 (3)	0.71^e^
Emergency admission, n (%)	64 (86.49%)	283 (90.71%)	0.28^b^
Need for intravenous antibiotics, n (%)	61 (82.43%)	159 (50.96%)	< 0.001^b^
Duration of antibiotics, in days, median (IQR)	12.5 (17)	1 (6)	< 0.001^e^
Duration of ER stay, in hours	36.13 (47)	37.75 (45.88)	0.73^e^
Duration of ICU stay, in hours, median (IQR)	0 (0)	0 (0)	0.03^e^
Need for invasive ventilation, n (%)	16 (21.62%)	39 (12.50%)	0.04^b^
Duration of invasive ventilation, in days, median (IQR)	0 (0)	0 (0)	0.01^e^
Need for non-invasive ventilation, n (%)	26 (35.14%)	55 (17.63%)	0.001^b^
Duration of non-invasive ventilation, in days, median (IQR)	0 (3)	0 (0)	0.0001^e^
Performance of procedure, n (%)	19 (25.68%)	81 (25.96%)	0.96^b^
Thoracentesis	7 (9.46%)	4 (1.28%)	0.001^d^
Central venous catheter insertion	8 (10.81%)	14 (4.49%)	0.048^d^
Performance of surgery, n (%)	24 (32.43%)	21 (6.73%)	< 0.001^b^
Underwent non-cardiac surgery, n (%)	23 (95.83%)	16 (76.19%)	0.08^d^
Surgical risk of non-cardiac surgeries, n (%)			0.03^d^
Low risk	1 (4.35%)	5 (31.25%)	
Intermediate risk	22 (95.65%)	11 (68.75%)	
Need for blood transfusion, n (%)	41 (55.41%)	99 (31.13%)	< 0.001^b^
In-hospital complications, n (%)	30 (40.54%)	56 (17.95%)	< 0.001^b^
Healthcare associated infections	21 (28.38%)	25 (8.01%)	< 0.001^b^
Respiratory complications	11 (14.86%)	13 (4.17%)	0.001^b^
Adverse drug events/adverse drug reactions	5 (6.76%)	5 (1.60%)	0.03^d^
Development of healthcare associated infections, n (%)	20 (27.03%)	25 (8.01%)	< 0.001^b^
Central line-associated bloodstream infection/catheter-related bloodstream infection	1 (1.35%)	0	0.19^d^
Hospital-acquired pneumonia	18 (24.32%)	24 (7.69%)	< 0.001^d^
Ventilator-associated pneumonia	5 (6.76%)	1 (0.32%)	0.001^d^
Number of comanaging services, n (%)	5 (3)	2 (3)	< 0.001^e^
Length of stay, in days, median (IQR)			
Overall	19 (19.5)	6 (6)	< 0.001^e^
Emergency admissions only	20.5 (8)	6 (5)	< 0.001^e^
Elective admissions only	3.5 (3)	2 (0)	< 0.001^e^
Length of stay, in days, mean difference (95% CI)			
Emergency admissions only	18 (95% CI 16.19, 19.85)		-
Elective admissions only	8 (95% CI 1.67, 14.42)		-

^a^computed using t-test

^b^computed using chi-square test

^c^due to missing data in some records

^d^computed using Fisher’s exact test

^e^computed using Mann–Whitney U test

As shown in Supplementary Table 3, positive predictors of PLOS include being partially dependent on admission (aOR 2.61, 95% CI 0.99, 6.86), more co-managing services (aOR 1.26, 95% CI 1.06, 1.50), and longer duration of intravenous antibiotics (aOR 1.36, 95% CI 1.22, 1.51). The only negative predictor is the need for intravenous antibiotics (aOR 0.14, 95% CI 0.04, 0.54).

Table 2 details the outcomes of the admissions with PLOS and NLOS. Outcome on the last day of hospitalization of admissions did not significantly differ between the two groups (*p*-value 0.813). Of the 74 admissions with PLOS, 9 expired (12.16%), 60 were discharged (81.08%), and 5 had unknown outcomes (i.e. home against medical advice, absconded, transfer to hospital of choice) (6.76%). Among those who expired, 3 died due to septic shock (33.33%), 2 from cardiogenic shock (22.22%), 2 from obstructive shock (22.22%), 1 from acute respiratory failure (11.11%), and 1 from fatal arrhythmia (11.11%).

**Table 2:** Outcomes of admissions with normal and prolonged length of stay

Variable	PLOS (*n* = 74)	NLOS (*n* = 312)	*P*-value
Outcome on last day of hospitalization, n (%)			0.81^a^
Home	60 (81.08%)	255 (81.73%)	
Mortality	9 (12.16%)	41 (13.14%)	
Unknown	5 (6.76%)	16 (5.13%)	
Immediate cause of death (n = 43)^b^			1.00^a^
Acute respiratory failure, n (%)	1 (11.11%)	5 (14.71%)	
Shock, n (%)	7 (77.78%)	22 (64.71%)	
Myocardial infarction, n (%)	0	3 (8.82%)	
Fatal arrhythmia, n (%)	1 (11.11%)	4 (11.76%)	
Hospitalization cost, median (IQR)	77,427.20 (102,596)	33,681.70 (57,601.47)	< 0.0001^c^

^a^computed using Fisher's exact test

^b^due to missing data in some records

^c^computed using Mann–Whitney U test

Around 68.9% of those with PLOS had missing data on reasons for delay in discharge. Of the 23 records with data, 12 had PLOS due to prolonged treatment (16.2%), 7 had delays in treatment (9.5%), 3 had delays in diagnostics (4.1%), and 1 developed in-hospital complications (1.4%). The median direct cost of hospitalization is significantly higher among patients with PLOS [PHP 77,427.20 (IQR 102,596) vs. PHP 33,681.70 (57,601.47), *p* < 0.0001] as shown in Table 2.

## Discussion

We found out that almost a fifth of emergency and a quarter of elective admissions had prolonged hospital stay. Positive predictors of PLOS include being partially dependent on admission, more co-managing services, and longer duration of intravenous antibiotics. The only negative predictor is the need for intravenous antibiotics. In those with available information, the most common reason for delay in discharge was prolonged treatment. The median hospitalization cost of patients with PLOS was PHP 77,427.20 (IQR 102,596), more than double of that with NLOS. Although there are several studies on PLOS involving medical and surgical admissions, this is the first study in a lower-middle income country that explored the prevalence and factors associated with PLOS among internal medicine patients.

The prevalence of PLOS in our study is higher than that of other studies. A study conducted in a 551-bed urban, quaternary-care academic medical center in the US had 2.3% of their discharges with PLOS while a nationwide study involving all hospitals of the Spanish Public Health Service reported 3.2% of discharges to have PLOS. They used > 21 days and > 30 days, as cut-off for PLOS, respectively [1, 2]. Other studies that involved admissions from various specialties also had lower prevalence of PLOS at 5.1% and 9.7% with cut-off of 34 days and 14 days for PLOS, respectively [4, 7]. We used a shorter cut-off for PLOS compared to the mentioned studies, which could explain the higher prevalence found in our study. Our findings also reflect the nature of the hospital as the national government referral center where patients with complex medical problems are brought from hospitals all over the country. Identified reasons for discharge delays such as delays in diagnostics and treatment which lead to PLOS, are also encountered in upper middle income and high income countries [11, 12].

Being partially dependent in terms of functional capacity on admission is associated with PLOS. This suggests debilitating, undertreated, or untreated comorbidities likely complicate management and adversely affect the patient’s response to treatment leading to prolonged hospital stay. These patients may also be sicker on admission predisposing them to a higher risk for in-hospital complications. A study on older hospitalized medical patients found that delayed discharge was associated with functional dependence [13]. Thyroid disease is found to be more common in those with PLOS possibly due to associated cardiovascular sequelae; this is similar to the findings of a study on patients with congestive heart failure who have hyperthyroidism [14].

During the course of their admission, patients with PLOS had a greater tendency to receive non-invasive and invasive ventilation, central venous catheters, and blood transfusions and undergo thoracentesis and surgeries. They also spent more days on non-invasive and invasive ventilation and intensive care unit (ICU) than those with NLOS. Altogether, these are surrogate markers for the complexities of the disease processes needing interventions which have complications of their own. Problems in weaning from the mechanical ventilator may lead to prolonged hospital stay while a longer stay in the ICU may indicate a more severe disease compounded with complications of management [5, 15].

In-hospital complications were found to be more common in those with PLOS. Healthcare-associated infections (HAI) such as hospital-acquired pneumonia extend length of stay due to need for intravenous antibiotics especially if no organism is isolated from cultures. A study of several hospitals in China found an increase of 10.4 days in patients who developed HAI [16]. Liberation from mechanical ventilation due to respiratory complications also add hospital days which can further predispose patients to more healthcare-associated infections such as ventilator-associated pneumonia. Adverse drug events or reactions are more common in those with PLOS. This is consistent with a study in a Nigerian Hospital that showed a significantly longer durations of hospital stay in those who developed adverse drug reactions compared to those who did not [17].

Having more co-managing specialty and subspecialty services is directly associated with longer hospital stay. While the primary service responsible for a patient acts as the final decision maker after considering inputs from other services, the occasionally contradicting opinions of services may delay patient management leading to prolonged hospital stay. Involvement of multiple specialties may also be indicative of complicated medical problems which may need longer hospitalization.

More days on intravenous antibiotics was also associated with prolonged hospital stay. Patients who were on intravenous antibiotics for a longer duration may have had more complicated, nosocomial infections with possible multidrug resistant organisms, necessitating longer hospital stays. On the other hand, the need for intravenous antibiotics was associated with less hospital days. The most plausible explanation for this finding is that those who have an acute infection as the main reason for admission only needed to complete a course of antibiotics to be eventually discharged. Typically, antibiotic treatment lasts 7 to 14 days with some patients possibly discharged on oral antibiotics after a few days of intravenous antibiotics to complete the antibiotic course on outpatient basis. In addition, the hospital has a Sepsis Pathway that likely contributed to the early administration of antibiotics for patients who are either admitted for sepsis or develop sepsis while admitted. These may have contributed to shorter hospital stay.

Prolonged treatment was the most common reason for delay in discharge similar to a study in a tertiary healthcare center in Mexico [4]. This correlates with the complex nature of the disease processes that requires further evaluation and management. However, the reason for delay was not documented in more than half of those with PLOS in our study, and thus robust conclusions cannot be drawn from this observation. Similar to a study among general internal medicine patients in a tertiary care center in Thailand, the median direct cost of hospitalization was significantly higher among patients with PLOS, thereby implying a greater economic burden for the hospital and strain on its resources [5]. In health systems with out-of-pocket payments as the dominant payment mechanism and social health insurance covering less than a fifth of total health expenditure, catastrophic health spending is worrisome. In 2012, 1 million Filipinos were impoverished by high out-of-pocket expenses [18].

We encountered several limitations in our study. First, the definition of PLOS varies in the literature, making comparisons across studies difficult. Another limitation is the retrospective nature of the study. Data were based on a review of handwritten chart entries, which may be limited by illegibility, incompleteness, or lack of proper documentation. The problem of missing data is particularly true for reasons for discharge delays wherein most of the reasons were not clearly stated or logged at all. Due to the relatively small sample size, some factors that were associated with PLOS in other studies may not have been statistically significant in this study. Our findings from a single public tertiary hospital may also not be generalizable to other healthcare institutions.

Given the results of the study, the government, in coordination with the hospital administration, should modify system-level factors such as lack of infrastructure and inadequate financing to address the health and financial impact of PLOS. Hospital policies should be reviewed and improved to mitigate risk factors and address reasons for discharge delays. Knowing that those who are partially dependent on activities of daily living on admission are at risk for PLOS, clinicians should pay attention to strategies on supporting patients with limited mobility and functionality such as a mobility bundle or improved coordination with rehabilitation service [19]. Interdepartmental communication should be improved through regular multidisciplinary meetings especially in patients with complex medical problems requiring multispecialty management. It is also recommended that an outpatient parenteral antimicrobial therapy (OPAT) program be developed to help address prolonged treatment as the most common reason for discharge delays. Finally, issues on missing data highlight gaps in documentation that should be addressed and included in quality control indices.

Additional research should be undertaken to create standard disease- or admission-specific definitions for PLOS as each condition entails different approaches to management with some requiring weeks of treatment. These definitions may allow comparisons across studies and serve as a metric for quality of care delivered. Furthermore, factors can be analyzed per type of admission (i.e. emergency, elective) to see if the associations are robust. Finally, interventions targeting factors that lead to PLOS should be conceptualized and tested in future studies.

## Conclusions

A significant proportion of admitted internal medicine patients have prolonged hospital stay. Various factors were found to be significant predictors. Knowledge of the factors associated with prolonged hospital stay and reasons for discharge delays can guide clinicians and hospital administrators in improving hospital efficiency and quality of patient care.

## Supplementary Information

**Additional file 1: Supplementary Table 1.** Study variables and their operational definitions. **Supplementary Table 2.** Other characteristics of admissions with normal and prolonged length of stay. **Supplementary Table 3.** Multiple logistic regression analysis of marginally associated variables.

## Data Availability

The datasets supporting the conclusions of this article are included within the article and its additional files.

## Abbreviations

HAI: Healthcare-associated infections
ICU: Intensive care unit
NLOS: Normal length of stay
OPAT: Outpatient parenteral antimicrobial therapy
PLOS: Prolonged length of stay
UP-PGH: University of the Philippines—Philippine General Hospital
