# Supplementary material for: Prevalence and predictors of prolonged length of stay among patients admitted under general internal medicine in a tertiary government hospital in Manila, Philippines: a retrospective cross-sectional study
Source: BMC Health Serv Res. 2023 Jan 18;23:50. doi: 10.1186/s12913-022-08885-4 (PMC9850543; doi:10.1186/s12913-022-08885-4)
Supplement: Supplementary file 1 — Additional file 1: Supplementary Table 1. Study variables and their operational definitions. Supplementary Table 2. Other characteristics of admissions with normal and prolonged length of stay. Supplementary Table 3. Multiple logistic regression analysis of marginally associated variables. [file 12913_2022_8885_MOESM1_ESM.docx]

SUPPLEMENTARY MATERIAL

**TABLE OF CONTENTS**

Supplementary Table 1. Study variables and their operational definitions p. 2-3

Supplementary Table 2. Other characteristics of admissions with normal and prolonged length of stay. p. 4-5

Supplementary Table 3. Multiple logistic regression analysis of marginally associated variables. p. 5

References p. 6-7

**Supplementary Table 1. Study variables and their operational definitions**

| **Variable** | **Operational definition** |
| --- | --- |
| Age | Age on admission in years as stated in the case record |
| Sex | Male or female as stated in the case record |
| Distance of place of residence from hospital | Distance of province from the hospital in kilometers estimated via Google Maps |
| Highest educational attainment | Highest educational attainment as stated in the case record or initial chart entry of the physician-on-duty or resident-in-charge of the Department of Medicine. |
| Employment status on admission | Employed or unemployed as stated in the personal and social history |
| Medical Social Service classification (A, B, C, or D) | Classification given by the Medical Social Service as stated in the case record |
| Smoking status (current, previous, or never)^1^ | Defined as current if the patient still smokes at the time of admission, previous if the patient quit at the time of admission, or never smoker if the patient never smoked as stated in the initial chart entry of the physician-on-duty or resident-in-charge of the Department of Medicine. |
| Level of alcohol consumption (heavy, occasional, or never)^2,3^ | Based on a standard drink of 14 g of pure alcohol which is roughly equivalent to 12 ounces of regular beer, 5 ounces of wine, or 1.5 ounces of distilled spirits. Defined as heavy if patients drank 4 or 5 drinks for women and men, respectively per day for 5 or more days in the past month; defined as occasional if patients drank less than the definition of heavy; defined as never if patients have never consumed alcoholic beverages, or as stated in the initial chart entry of the physician-on-duty or resident-in-charge of the Department of Medicine. |
| Functional status on admission (independent, partially assisted, or dependent)^4^ | Defined as independent or very dependent on activities of daily living according to the Katz Index of Independence in Activities of Daily Living or as stated in the initial chart entry of the physician-on-duty or resident-in-charge of the Department of Medicine. |
| Admission type (elective or emergency)^5^ | Elective is defined as pre-arranged, non-emergency admissions with an appointment date while emergency is defined as admissions for patients with urgent or life-threatening conditions that require immediate medical assistance. |
| Day of admission (weekday or weekend) | Weekday defined as Monday to Friday; weekend defined as Saturday or Sunday. Admissions during declared regular and special non-working holidays will be classified as weekend admissions. |
| Time of admission (within or beyond office hours) | The official time of admission will be as stated in the case record. Within office hours defined as 7:00 am to 4:30 pm or 11:30 am for weekdays and weekends or holidays, respectively; beyond office defined as 4:31 pm or 11:31 pm to 6:59 am on the following day for weekdays and weekends or holidays, respectively. |
| Prior hospitalization in the past 30 days | Includes admission at the Philippine General Hospital or other hospitals. |
| Comorbidities on admission | Comorbidities of the patient as stated initial chart entry of the physician-on-duty or resident-in-charge of the Department of Medicine. This is limited to the following: hypertension, type 2 diabetes mellitus, ischemic heart disease, congestive heart failure, stroke, myocardial infarction, bronchial asthma, chronic obstructive pulmonary disease, past pulmonary tuberculosis, thyroid disease, chronic kidney disease, malignancy, human immunodeficiency virus/acquired immunodeficiency syndrome (HIV/AIDS), chronic liver disease, hepatitis, gouty arthritis, osteoarthritis, rheumatoid arthritis, peptic ulcer disease |
| Number of comorbidities | Comorbidities of the patient as stated in the initial chart entry of the physician-on-duty or resident-in-charge of the Department of Medicine. |
| Charlson Comorbidity Index on admission^6^ | A prognostic scoring system for 10-year survival using the following variables: age, myocardial infarction, congestive heart failure, peripheral vascular disease, cerebrovascular accident or transient ischemic attack, dementia, connective tissue disease, peptic ulcer disease, liver disease, diabetes mellitus, hemiplegia, moderate to severe chronic kidney disease, solid tumor, leukemia, AIDS |
| Number of medications on admission | Number of medications on admission as ordered by the physician-on-duty or resident-in-charge of the Department of Medicine. |
| Need for intravenous antibiotics and duration | Use of intravenous antibiotics as stated in the therapeutic sheet and total duration in days of all antibiotics used. |
| Duration of stay in the emergency department | Number of hours of stay in the emergency department counted from the day of admission as stated in the case record to the day that the patient was physically transferred to the ward or intensive care unit. This is not applicable for elective admissions. |
| Duration of stay in the intensive care unit | Number of days of stay in the intensive care unit counted from the day that the patient was physically transferred to the medical intensive care unit to the day that the patient was physically discharged or transferred to the ward. |
| Need for invasive ventilation and duration | Use of a mechanical ventilator and duration in days |
| Need for non-invasive ventilation and duration | Use of a bilevel positive airway pressure or continuous positive airway pressure machine and duration in days |
| Performance of surgery or procedure^7^ | Number and type of surgery or procedure done. Surgeries will be categorized as cardiac or noncardiac surgeries. Non-cardiac surgeries will be further classified as high risk, intermediate risk, and low risk as defined by the American College of Cardiology/American Heart Association. For procedures, it will include thoracentesis, paracentesis, central venous catheter insertions, and biopsies. |
| Need for blood transfusion | Transfusion of blood products as stated in the transfusion flow sheet |
| Need for dialysis | Diagnosis of acute kidney injury or chronic kidney disease requiring hemodialysis or peritoneal dialysis |
| Development of shock and duration | Diagnosis of shock irrespective of type and total duration of vasopressor in days |
| In-hospital complications^8–10^ | Development of new medical problems after admission deemed as harmful events resulting from the process of care and treatment rather than the natural progression of disease such as pressure injuries, falls, healthcare-associated infections (HAIs), surgical complications, respiratory complications, cardiac complications, venous thromboembolism, renal failure, gastrointestinal bleeding, and adverse drug events. HAIs will be further classified into specific types such as central-line associated bloodstream, catheter-associated urinary tract infection, hospital-acquired pneumonia, ventilator-associated pneumonia, and surgical site infections. HAIs diagnosed at the time of admission are excluded and are presumed to have incubated during the previous admission. |
| Number of co-managing services | Number of services from other subspecialties or departments that made at least one chart entry |
| Signed advance directive | Presence of a signed advance directive form or a chart entry with statements pertaining to advance directives such as “Do not intubate”, “Do not resuscitate”, “Discontinue vasopressors”, “Discontinue medications”, “No labs”, “No intravenous fluids”, “No blood transfusion”, and “No hemodialysis”. A retracted advance directive will not be counted. |
| Outcome of hospitalization | Defined as discharged if the patient was cleared by the physician to leave the hospital after receiving inpatient care.  Defined as expired if the the patient died during admission  Defined as home against medical advice if the patient decided to leave the hospital against the advice of the physician  Defined as absconded if the patient left the hospital without informing any of the hospital staff |
| Cause of death | Immediate cause of death as written in the Death Certificate. |

**Supplementary Table 2. Other characteristics of admissions with normal and prolonged length of stay.**

| **Variable** | **PLOS**  **(n = 74)** | **NLOS**  **(n = 312)** | **P-value** |
| --- | --- | --- | --- |
| Highest educational attainment (N = 80)^a^ | | | 0.32^b^ |
| Elementary, n (%) | 6 (28·57%) | 7 (11·67%) |  |
| High school, n (%) | 13 (61·90%) | 43 (71·67%) |  |
| Technical vocational, n (%) | 0 | 1 (1·67%) |  |
| College, n (%) | 2 (9·52%) | 8 (13·56%) |  |
| Distance of place of residence from hospital, in kilometers, median (IQR) | 50·6 (47·2) | 50·6 (47·2) | 0.11^c^ |
| Medical Social Service Classification (N = 217)^a^ | | | 1.00^d^ |
| A, n (%) | 0 | 3 (1·71%) |  |
| C, n (%) | 1 (2·22%) | 5 (2·91%) |  |
| D, n (%) | 44 (97·78%) | 164 (95·35%) |  |
| Comorbidities | | | |
| Previous stroke, n (%) | 4 (5·41%) | 14 (4·49%) | 0.76^d^ |
| Previous myocardial infarction, n (%) | 1 (1·35%) | 11 (3·53%) | 0.48^d^ |
| Chronic obstructive pulmonary disease, n (%) | 0 | 2 (0·64%) | 1.00^d^ |
| Past pulmonary tuberculosis, n (%) | 11 (14·86%) | 46 (14·74%) | 0.98^b^ |
| HIV/AIDS, n (%) | 0 | 3 (0·94%) | 0.53^d^ |
| Gouty arthritis, n (%) | 1 (1·35%) | 6 (1·92%) | 1.00^d^ |
| Chronic Liver Disease, n (%) | 1 (1·35%) | 6 (1·92%) | 1.00^d^ |
| Hepatitis, n (%) | 0 | 6 (1·92%) | 0.60^d^ |
| Osteoarthritis, n (%) | 1 (1·35%) | 6 (1·92%) | 0.10^d^ |
| With prior hospitalization in the past 30 days, n (%) | 20 (27·03%) | 65 (20·83%) | 0.25^b^ |
| Weekday admission, n (%) | 57 (77·03%) | 236 (75·64%) | 0.80^b^ |
| Admitted beyond office hours, n (%) | 39 (52·70%) | 199 (63·78%) | 0.08^b^ |
| Number of medications, median (IQR) | 7·5 (4) | 6 (5) | 0.07^c^ |
| Performance of procedure, n (%) | | | |
| Paracentesis | 0 | 4 (1·28%) | 1.00^d^ |
| Biopsies | 1 (1·35%) | 15 (4·81%) | 0.33^d^ |
| Endoscopy | 3 (4·05%) | 16 (5·13%) | 1.00^d^ |
| Colonoscopy | 0 | 2 (0·64%) | 1.00^d^ |
| Coronary angiography | 1 (1·35%) | 14 (4·49%) | 0.32^d^ |
| Percutaneous coronary intervention | 2 (2·70%) | 13 (4·17%) | 0.75^d^ |
| Bronchoscopy | 0 | 2 (0·64%) | 1.00^d^ |
| Need for dialysis, n (%) | 15 (20·27%) | 41 (13·14%) | 0.12^b^ |
| Duration of shock, in days, median (IQR) | 0 (0) | 0 (0) | 0.64^c^ |
| Type of shock, n (%) | | | |
| Septic | 9 (12·16%) | 42 (13·46%) | 0.77^b^ |
| Cardiogenic | 5 (6·76%) | 24 (7·69%) | 0.78^b^ |
| Hypovolemic | 2 (2·70%) | 10 (3·21%) | 1.00^d^ |
| Obstructive | 3 (4·05%) | 5 (1·60%) | 0.18^d^ |
| In-hospital complications, n (%) | | | |
| Pressure injuries | 3 (4·05%) | 4 (1·28%) | 0.13^d^ |
| Cardiac complications | 1 (1·35%) | 5 (1·60%) | 1.00^d^ |
| Venous thromboembolism | 2 (2·70%) | 1 (0·32%) | 0.10^d^ |
| Renal failure | 9 (12·16%) | 22 (7·05%) | 0.15^d^ |
| Gastrointestinal bleeding | 3 (4·05%) | 7 (2·24%) | 0.41^d^ |
| Advance directive, n (%) | 7 (9·46%) | 32 (10·06%) | 0.88^b^ |

^a^due to missing data in some records

^b^computed using chi-square test

^c^computed using Mann-Whitney U test

^d^computed using Fisher’s exact test

**Supplementary Table 3. Multiple logistic regression analysis of marginally associated variables.**

|  | **Adjusted odds ratio**  **(95% Confidence Intervals)** | **P-value** |
| --- | --- | --- |
| Function (partially dependent vs. independent) | 2.61 (0.99-6.86) | 0.052 |
| Number of comanaging services | 1.26 (1.06-1.50) | 0.008 |
| Need for intravenous antibiotics | 0.14 (0.04-0.54) | 0.004 |
| Duration of antibiotics | 1.36 (1.22-1.51) | <0.001 |

**REFERENCES**

1 Center for Disease Control and Prevention. NHIS - Adult Tobacco Use. Centers for Disease Control and Prevention; 2017 Aug 29. Available from: <https://www.cdc.gov/nchs/nhis/tobacco/tobacco_glossary.htm>. Accessed 16 July 2020.

2 National Institute on Alcohol Abuse and Alcoholism. What is a standard drink? National Institutes of Health. Date unknown. Available from: <https://www.niaaa.nih.gov/what-standard-drink>. Accessed 16 July 2020.

3 National Institute on Alcohol Abuse and Alcoholism. Drinking levels defined. National Institutes of Health. Date unknown. Available from: https://www.niaaa.nih.gov/alcohol-health/overview-alcohol-consumption/moderate-binge-drinking. Accessed 16 July 2020.

4 Edemekong PF, Bomgaars DL, Sukumaran S, Levy SB. StatPearls. StatPearls Publishing. 2020. Available from:

<https://www.ncbi.nlm.nih.gov/books/NBK470404/>. Accessed 16 July 2020.

5 Krämer J, Schreyögg J, Busse R. Classification of hospital admissions into emergency

and elective care: a machine learning approach. Health Care Manag Sci

2019; doi:10.1007/s10729-017-9423-5.

6 Charlson ME, Pompei P, Ales KL, MacKenzie CR. A new method of classifying

prognostic comorbidity in longitudinal studies: development and validation. J Chronic

Dis 1987; doi:10.1016/0021-9681(87)90171-8.

7 Fleisher LA, Beckman JA, Brown KA, et al. ACC/AHA 2007 guidelines on

perioperative cardiovascular evaluation and care for noncardiac surgery: a report of

the American College of Cardiology/American Heart Association Task Force on

Practice Guidelines (Writing Committee to Revise the 2002 Guidelines on

Perioperative Cardiovascular Evaluation for Noncardiac Surgery): developed in

collaboration with the American Society of Echocardiography, American Society of

Nuclear Cardiology, Heart Rhythm Society, Society of Cardiovascular

Anesthesiologists, Society for Cardiovascular Angiography and Interventions, Society

for Vascular Medicine and Biology, and Society for Vascular Surgery [published

correction appears in Circulation. 2008 Aug 26;118(9): e143–4] [published correction

appears in Circulation. 2008 Feb 5;117(5):e154]. Circulation 2007; doi:

10.1161/CIRCULATIONAHA.107.185699

8 Lagoe RJ, Westert GP. Evaluation of hospital inpatient complications: a planning

approach. BMC Health Serv Res 2010; doi:10.1186/1472-6963-10-200

9 Australian Commission on Safety and Quality in Health Care. Hospital-Acquired

Complications Fact Sheets. Australian Commission on Safety and

Quality in Health Care. 2018. Available from: https://www.safetyandquality.gov.au/sites/default/files/migrated/Short-Hospital-Acquired-Complications-Factsheets-all-HACs.pdf. Accessed 16 July 2020.

10 Hughes JS, Averill RF, Goldfield NI, et al. Identifying potentially preventable

complications using a present on admission indicator. Health Care Financ Rev

2006;27:3.
